# Characterizing passenger-ship emissions: towards improved sustainability for MedMar fleet (gulf of Naples)

**DOI:** 10.1007/s12053-022-10064-7

**Published:** 2022-10-18

**Authors:** Antonio Spagnuolo, Giuseppe De Santo, Carmela Vetromile, Antonio Masiello, Pierluigi Di Costanzo, Salvatore Esposito, Umberto Buono, Maria Rosa di Cicco, Carmine Lubritto

**Affiliations:** 1 Department of Environmental Biological and Pharmaceutical Sciences and Technologies, University of Campania “L. Vanvitelli”, Via A. Vivaldi 43, Caserta, Italy; 2 Energreenup S.r.l., Via Acquaviva 192, Caserta, Italy; 3 MedMar Navi S.p.A., Via Roma 1, Pozzuoli, Italy

**Keywords:** Cargo capacity, Fuel consumption, Navigation speed, Passenger ships, Shipping emissions, Simulations

## Abstract

Promoting energetic and environmental sustainability in the naval sector requires a necessary understanding of the energy demand of vessels and of the factors affecting it. This article shows the results of a study conducted by the shipping company MedMar aimed at acquiring a detailed analysis of the energetic performances of its fleet. The study involved the analysis of fuel consumption and emissions of the fleet using a specific software and under different scenarios, assuming the navigation speed and the cargo level of the vessels as reference parameters. Simulations also provided a comparison, concerning emissions and externalities, between ships and two different means of transport. The purpose of this study was to identify potential areas of improvement, where ad hoc strategies could be used to further optimise the energetic and environmental performance of MedMar fleet and mitigate its impact on the delicate ecosystem of the gulf of Naples, where the fleet sails.

**Supplementary Information:**

The online version contains supplementary material available at 10.1007/s12053-022-10064-7.

## Introduction

Climate change and its consequences are currently of great concern, across all manufacturing sectors and human activities (Goldsworthy and Goldsworthy 2015; Marelle et al. 2016; Song 2014). The shipping and marine sector, which accounts for about 90% of trade-related traffic (Brenna et al. 2020), also features among the activities addressed by the scientific community (Nunes et al. 2020; Yan et al. 2017). While shipping is currently the most efficient transport system in terms of emissions per amount of goods transported and distances covered (Brenna et al. 2020), the consumption of fuel accounts for a significant portion of the operating costs of a vessel, and one of the most critical issues in the sector is the compliance with acceptable limits in terms of harmful gas emissions from combustion engines (Hansen et al. 2020; López-Aparicio et al. 2017; Zis and Psaraftis 2017, 2019). As pointed out by Lehmann et al. (2021), the COVID-19 outbreak triggered a joint reaction by the world’s leading governments to encourage the transition of industrialised societies towards environmental sustainability, in the perspective of “rebuilding better”. In the race towards greater sustainability, the maritime sector also plays a key role, and implementing strategies to reduce its associated consumption is one of the major current challenges (Dewan et al. 2018; Rehmatulla et al. 2017), especially considering the strong pressure on the fossil fuel market triggered by the global economic upturn subsequent to the most acute phases of the COVID-19 pandemic (Drăgoi and Bâlgăr 2021).

Significantly, if the shipping sector was considered as a distinct country, it would rank as the sixth largest emitter of greenhouse gases (GHG) in the world, right between Japan and Germany. The maritime sector is significantly responsible for a large part of emissions of marine pollutants (substance release, thermal and noise pollution) and atmospheric pollutants (NOx, SOx, PM) (Brenna et al. 2020), while its share of GHG emissions currently corresponds to 2–3% on a global scale (Smith et al. 2014), exceeding or almost equalling many other sectors that provide services of extreme importance to humans, such as the water (di Cicco et al. 2019), waste stream treatment (di Cicco et al. 2021) and telecommunications sectors (Vetromile et al. 2021). But this number could still increase, because the demand for maritime transport is growing rapidly and so are the associated fuel consumption emissions. Abramov and Abramov (2021), based on statistical data covering the period 2000–2018, estimated growth rates of marine transport volumes for the quinquennium 2019–2023 ranging from 1.5 to 3.8% on a global scale. Mersin et al. (2020) reported that the maritime sector will be responsible for about 15% of global greenhouse gas emissions by 2050. In the Fourth International Maritime Organisation (IMO) GHG Study of 2020 (available at: https://wwwcdn.imo.org/localresources/en/OurWork/Environment/Documents/Fourth IMO GHG Study 2020 - Full report and annexes.pdf, accessed on 22/09/2022), on the other hand, projections based on long-term socio-economic pathways predict that maritime transport emissions will increase from 1000 Mt CO_2_ in 2018 to 1000–1500 Mt CO_2_ in 2050, reflecting the estimated increase in world population to 9.9 billion over the same period (2021 World Population Data Sheet, available at: https://interactives.prb.org/2020-wpds/, accessed on 04/02/2022).

But not only the navigation itself has a strong impact, in fact, a significant part of the atmospheric emissions of the shipping sector is also due to port docking (Liu et al. 2018; Song 2014), while the permanence of ships in the port area poses a high risk to human health and has a huge cost for the community (Corbett et al. 2007; Liu et al. 2016; Nunes et al. 2019).

For all these reasons, in recent years, there has been a surge of scientific studies to characterise the energy consumption and environmental impact of naval fleets, aimed at proposing descriptive models of their performance and implementing effective strategies to lower operating costs. Mersin et al. (2020), for example, focused on the simulation of how to potentially reduce greenhouse gas emissions from cargo ships by acting on the cruising speed. According to the literature, reducing the speed leads to a reduction in fuel consumption and associated emissions, thereby increasing the energetic and environmental performances of the vessel (Cariou 2011; Corbett et al. 2009; Tillig et al. 2020). Im et al. (2019) proposed a method for assessing the energy efficiency of ship fleet operating conditions based on the Ship Energy Efficiency Management Plan (SEEMP) guidelines, introduced by the IMO at the 59th meeting of the Marine Environment Protection Committee. Furthermore, Chi et al. (2018) implemented and proposed a software for monitoring the energy efficiency index of marine vessels, through which it is possible to check and monitor in real time both the fuel consumption and related emissions of an individual vessel.

As in many other industrial contexts, including for example the water sector, one of the main obstacles to the implementation of such methodologies is the frequent unavailability of consumption and emission data, on whose basis analyses can be carried out to achieve energy optimisation goals (di Cicco et al. 2020a, 2020b; Johansson et al. 2017; Moreno-Gutiérrez et al. 2015; Toscano and Murena 2019). As a result, one of the strategies currently taken by international governmental authorities is to provide directives for the shipping sector aimed at implementing the monitoring and auditing of vessels’ emissions and their overall performance. One of the first initiatives was taken precisely in 2014 by the IMO, which approved a set of mandatory requirements to record and report fuel consumption by naval fleets, in order to create an energy efficiency framework (Karim 2016). Subsequently, in 2015, the European Parliament and the Council proposed a new strategic method known as “MRV” (EU Regulation 2015/757) for “Monitoring, Reporting and Verification” of carbon dioxide emissions from maritime transport. The objective of the EU MRV Regulation was to create a new legal framework for collection of data on maritime transport emissions; such data about ship efficiency would be provided to relevant markets with the aim of developing further policies towards greater sustainability and encourage emission reductions (Boviatsis and Tselentis 2019). Specifically, the EU MRV Regulation compelled companies to “monitor, report and verify” on an annual basis (starting from 2018) the fuel consumption, CO_2_ emissions and energy efficiency of their ships on voyages to and from European Economic Area (EEA) ports (Doundoulakis and Papaefthimiou 2022; Eftestøl and Yliheljo 2022). Subsequently, on 1/03/2018, a new regulation released by IMO became effective, wherein basically it was proposed the “DCS” (“Data Collection System”) method for fuel consumption data management in 3 steps, namely, data collection, data analysis and, if appropriate, deciding on measures to be adopted to improve performance (Boviatsis and Tselentis 2019). With this new regulation, IMO adopted an obligation already foreseen in MARPOL Annex VI, namely, that ships must record and report their fuel oil consumption on an annual basis, as stated in Resolution MEPC.278(70) (Adamowicz 2022; Deling et al. 2020).

The present work falls within this context, presenting the results of a research on the consumptions and related emissions of the company MedMar S.p.A., performed with the aim of estimating and studying the efficiency of their environmental performance. While increasing awareness on the energetic and environmental performance of its fleet, the main purpose of the shipping company was to acquire a tool for a better planning of future interventions that could mitigate the impact of the fleet on the ecosystem of the gulf of Naples, as well as contributing to the achievement of sustainability objectives. This work was also designed to provide useful data for comparison with the international scientific community which, as pointed out in the previous paragraphs, is one of the priority objectives to be pursued in this context, as established by international institutional authorities.

## Materials and methods

### Description of the company

MedMar Navi S.p.A. is a company founded in Naples in 1969 which operates in the maritime transport sector. Every year, the company provides around 9,000 connections, transporting over 1,800,000 passengers and 450,000 vehicles. The company also handles the maritime transport of automotive fuels, gas for heating and for civil use and a wide range of special and non-special waste, using dedicated vessels. Over the years, the company gradually took on a leading role in transport and logistics in the gulf of Naples, which is one of the most important tourist destinations in Campania and the whole of southern Italy, and which most needs environmental and ecosystem preservation against pollution from maritime traffic, especially in the port area (Mercogliano et al. 2016; Sciarrillo et al. 2020).

MedMar’s fleet comprises 7 vessels with different technical characteristics, performance, and cargo capacity. Specifically, the vessel ID names are MedMar Giulia; Benito Buono; Maria Buono; Rosa D’Abundo; Quirino; Tourist Ferry Boat Terzo; and Agata. Table 1 provides a summary of all the main details concerning these vessels. Data reported in Table 1 were collected and used for simulation purposes, as it will be more deeply discussed in the following sections.

**Table 1:** Summary of the main technical-geometric, load and speed data for each of the seven vessels belonging to the MedMar fleet. Supplementary data were provided in Table S1.

**Name of the ship**	**Draught (m)**	**Gross tonnage** **(t)**	**Maximum deadweight tonnage (t)**	**Maximum length** **(m)**	**Maximum width** **(m)**	**Total engine power** **(kw)**	**No. passengers** **(max)**	**No. cars** **(max)**	**Maximum speed** **(kn)**
**MedMar Giulia**	4.2	4833	711	86	16	4120	786	180	16.5
**Benito Buono**	3.8	2303	610	74	17	2352	800	90	13.5
**Maria Buono**	3.9	2543	949	84	15	4412	800	100	18.0
**Rosa D’Abundo**	3.4	844	390	70	13	3412	650	50	17.0
**Quirino**	3.4	1476	609	70	14	3706	620	50	16.0
**Tourist Ferry Boat Terzo**	2.5	438	212	58	9	1280	295	30	12.5
**Agata**	3.5	1310	1088	73	16	2984	400	92	13.5

### Overview of the methodology used for data analysis

The aim of the study was to determine, through simulations of different scenarios, vessels’ consumptions and associated pollutant emissions. The work proceeded in five stages:

1. Collection of the real data that were required by the computational model used in the study, i.e. geometric and structural data, engine power and maximum load (data shown in Table 1).
2. Collection of data regarding the real fuel consumptions of the vessels over a period of three years (from 2017 to 2019), in order to compare real data with those resulting from the simulations and, in this way, verifying the reliability of such simulations.
3. Choice of scenarios to simulate emissions from the main engines under different system and navigation parameters.
4. Choice of scenarios to simulate emissions from auxiliary services.
5. Choice of a reference scenario for the calculation of externalities and comparison with two other types of transport vehicles (car, bus).

Concerning emissions in a narrow sense, the pollutants considered for the simulations are those fundamental to the CEN 16258 standard, namely, SOx, NOx, CO2, CO, hydrocarbons and particulate matter. These substances are of specific interest because each of them contributes selectively to (i) oxygen depletion in inland and coastal waters, (ii) atmospheric ozone depletion and ground-level ozone creation, (iii) acidification of rainfall, (iv) accumulation of heavy metals and PCBs in the food chain and (v) eutrophication (Brenna et al. 2020). According to CEN 16258, for the calculation of total ship emissions due to passenger and freight transport, there are mainly two different allocation methods, namely, the mass method and the area method; for the present study, the mass allocation method was chosen.

In order to calculate the consumption and emissions of each vessel, it was decided to take into account two separate contributions: one related to the propulsion part (main engines) and one related to the auxiliary services (diesel/generators). The variables chosen as reference parameters in the three scenarios described below were mainly the navigation speed and the cargo levels of the vessels. Subsequently, the results obtained for the two individual contributions (main engines and diesel/generators) were compared and correlated to assess the average daily emissions.

### Scenarios and parameters used for the simulations

#### Simulations on the main engines

A calculation software named SHIP-DESMO-Ro-Ro Passenger was taken as a reference for the calculation of fuel consumption and emissions of main engines. This software was developed as part of a research project called RoRoSECA (RoRo-SECA project 2017), which was funded by DMF (Danish Maritime Fund) and that operated between 15/6/2015 and 14/6/2017; during this period, it was led by the Department of Transport until 30/4/2016 and by the Department of Management Engineering later on. The modelling tool was developed from extensive regression analysis of primary data from hundreds of Ro-Ro vessels operating in the Nordic Area, and it allows to obtain fuel consumption and other output data based on a relatively limited amount of input information. In view of its computational features, the software was written and optimised for larger ships than those belonging to the MedMar fleet (RoRo-SECA project 2017; Zis et al. 2017). For this reason, the model and the simulation software had to be modified, adapted, and rewritten so that it could conform as much as possible to the technical and structural requirements of the fleet under study.

##### Scenario no. 1.

In order to estimate pollutant emissions during navigation, the simulations were carried out assuming a reference scenario for the whole MedMar fleet. This choice was made considering the estimated average annual load of the fleet as well as to have homogeneous comparisons of results. Scenario no. 1 assumed as standard the following parameters:

- Deadweight tonnage (hereafter DT) at 75% of maximum load.
- Average vessel speed (this is based on navigation data available from www.marinetraffic.com, accessed on 04/02/2022).
- Technical/geometric parameters as designed for each vessel.
- With this basis, Table 2 shows the values that were assumed for each vessel starting from the real technical data reported in Table 1.

**Table 2:** Reference scenarios data for each vessel. The utilised deadweight tonnage corresponds to 75% of the maximum value reported in Table 1.

	**Scenario 1**		**Scenario 2**				**Scenario 3**			
**Name of the ship**	**Utilised DT (t)** 75% of DT max	**Navigation speed (kn)** medium value	**Total utilised DT (t)** 40% of DT max + total weight of passengers and vehicles	**Navigation speed (kn)** maximum value	**No. passengers** 50% of no. max	**No. vehicles** 50% of no. max	**Total utilised DT (t)** 40% of DT max + total weight of passengers and vehicles	**Navigation speed (kn)** maximum value	**No. passengers** 100% of no. max	**No. vehicles** 100% of No. max
**MedMar Giulia**	533	13.5	498	16.5	393	90	674	16.5	786	180
**Benito Buono**	458	13.0	353	13.5	400	45	459	13.5	800	90
**Maria Buono**	712	11.0	505	15.5	400	50	619	15.5	800	100
**Rosa D’Abundo**	293	11.5	227	17.0	325	25	297	17.0	650	50
**Quirino**	457	14.0	317	16.0	310	25	382	16.0	620	50
**Tourist Ferry Boat Terzo**	159	10.0	132	12.5	146	15	166	12.5	295	30
**Agata**	816	12.0	501	13.5	200	46	591	13.5	400	92

Remaining within the same scenario, and since the dependence of consumption and relative emissions on speed is documented in most scientific literature (inter alia Corbett et al. (2009); Tillig et al. (2020); Wiesmann (2010)), with the aim of confirming this dependence, a series of simulations were carried out to calculate the savings in terms of consumption and emissions when varying the speed. In particular, starting from the parameters indicated for scenario no. 1 (Table 2), the consumption of each ship was simulated at speeds gradually reduced by (i) half a knot, (ii) one knot and (iii) two knots, comparing to the average measured speed.

##### Scenarios no. 2 and no. 3.

Lastly, two other scenarios were analysed in relation to propulsion, in order to study the dependence of fuel consumption (and related emissions) on the amount of cargo embarked, in terms of passengers and cars. In these two scenarios, the amount of cargo on board was allocated as follows:

- Scenario (2): 50% of passengers and 50% of cars compared to the maximum cargo capacity.
- Scenario (3): 100% of passengers and 100% of cars compared to the maximum cargo capacity.

Regarding other parameters, the speed of the vessels was set equal to their maximum design speed in both scenarios, in order to simulate and evaluate the dependence of consumptions only on variable weight. In addition, it was assumed that 40% of the maximum DT for each vessel in the fleet was already on board before the embarkation of passengers and cars. Moreover, to approximate the weight related to passengers and cars on board, the following assumptions were applied: a single passenger was assigned an average weight of 100 kg, accounting for the presence of any possible luggage; a single car was assigned an average weight of 1500 kg. Table 2 summarises the starting parameters of the simulations for cargo scenarios 2 and 3 here illustrated.

#### Simulations on auxiliary services (diesel/generators)

Ships use diesels/generators (hereafter DG) to meet auxiliary services on board, which are needed during both phases of navigation and stationing in port (“port docking”). Each vessel had a number of DG, whose functioning varied according to the operational status of the vessel, and the different percentage of cargo. Table 3 summarises in detail the number of DG and the relative cargo levels for each of the vessels both in port and in navigation, as gathered from monitoring activities performed on the vessels. The company also provided, for each vessel, information on the real consumptions of the entire DG compartment and the nominal powers of each DG apparatus, useful for conducting simulations for this sector.

**Table 3:** Number of DG in relation to the load carried by each vessel, both in navigation conditions and when stationed in port.

**Name of the ship**	**Ship in port docking**	**Ship in navigation phase**
**MedMar Giulia**	No. 2 DG, 70% of the cargo	No. 4 DG, 80% of the cargo
**Benito Buono**	No. 1 DG, 80% of the cargo	No. 2 DG, 80% of the cargo
**Maria Buono**	No. 1 DG, 80% of the cargo	No. 2 DG, 80% of the cargo
**Rosa D’Abundo**	No. 1 DG, 80% of the cargo	No. 2 DG, 80% of the cargo
**Quirino**	No. 2 DG, 80% of the cargo	No. 3 DG, 80% of the cargo
**Tourist Ferry Boat Terzo**	No. 1 DG, 60% of the cargo	No. 2 DG, 60% of the cargo
**Agata**	No. 1 DG, 60% of the cargo	No. 2 DG, 60% of the cargo

In order to simulate and quantify the emissions of the DG, it was assumed that DG units run at a fixed number of rotations and considering that the cargo levels during both the navigation and port phases was different. These estimates were partly validated by a check carried out by engineers on the vessels here under study.

## Results and discussion

**Fig. 1:**
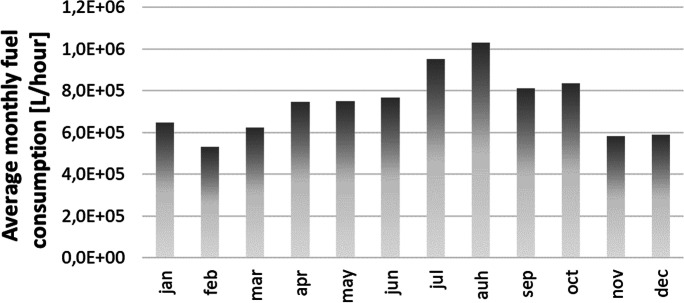
Average monthly fuel consumptions of MedMar naval fleet

From an analysis of the real data on fuel usage collected during the on-site surveys, it was possible to evaluate that the MedMar company currently operates with an average annual fuel consumption of about 8.800.000 litres. Figure 1 provides a plot of the average monthly fuel usage over a period of three years (2017–2019), for which all the data were available at the time the surveys took place. Given the type of fuel used by the company (MDO) and that the CO_2_ equivalent emissions are proportional to the fuel oil consumption, following the specific emission rates for each fuel reported by Buhaug et al. (2009), the consumptions of the company could be translated into annual CO_2_ equivalent emissions of about 2000 t.

### Emissions of the main engines

#### Scenario no. 1

Table 4 shows the results of the simulations performed for each of the vessels in the company’s fleet concerning the propulsive units (engines) during the navigation phase. The pollutants being considered are those reported in the “Overview of the methodology used for data analysis” section. Table 4 also shows the main engines fuel consumption of each vessel expressed as litres per hour and actually measured by MedMar company operators.

**Table 4:** Summary of estimated emissions for each vessel based on the reference scenario defined above; “fuel consumption” are real values effectively measured by MedMar technical staff.

Name of the ship	Fuel consumption (L/h)	Emissions					
		CO_2_ (t/h)	NO_x_ (kg/h)	SO_x_ (kg/h)	CO (kg/h)	HC (kg/h)	Particulate (kg/h)
**MedMar Giulia**	672.08	1.70	26.01	1.11	1.35	1.35	0.73
**Benito Buono**	491.33	1.24	19.02	0.81	0.99	0.99	0.53
**Maria Buono**	486.33	1.23	18.82	0.81	0.98	0.98	0.53
**Rosa D’Abundo**	259.18	0.66	10.03	0.43	0.52	0.52	0.28
**Quirino**	552.98	1.40	21.61	0.92	1.13	1.13	0.61
**Tourist Ferry Boat Terzo**	135.29	0.34	5.24	0.22	0.27	0.27	0.15
**Agata**	369.78	0.94	14.31	0.61	0.75	0.75	0.40

As mentioned in the “Overview of the methodology used for data analysis” section, an initial check on the reliability of the simulation data was carried out by comparing the simulated fuel consumptions with the real ones. To make this comparison, the simulated fuel consumption of each vessel (Table 4) was multiplied by the real navigation hours of each vessel, which were obtained from an online free database that tracks in real-time and keeps a record of the routes and sailing frequency of ships worldwide (available at https://www.vesselfinder.com/it, accessed on 04/02/2022). The comparison between simulated and real consumptions provided a difference within a 10% range, which can be considered a good level of approximation and reliability for the simulated data.

At the end of the “Overview of the methodology used for data analysis” section, it was mentioned that the main variables used as reference parameters for the proposed scenarios were navigation speed and cargo levels. When studying the possible correlations between the estimated emissions and all other parameters, it emerged that emissions, besides the logical dependence on fuel consumption (Pearson’s correlation index, *ρ* > 0.99), were indeed correlated both to the amount of cargo transported by the ships and, most importantly, to the average speed at which the ships travel. In particular, the study of Pearson’s correlation index returned a moderate correlation (0.51 < *ρ* < 0.52) between emissions and cargo transported, and a strong correlation (0.80 < *ρ* < 0.81) between emissions and average speed.

**Fig. 2:**
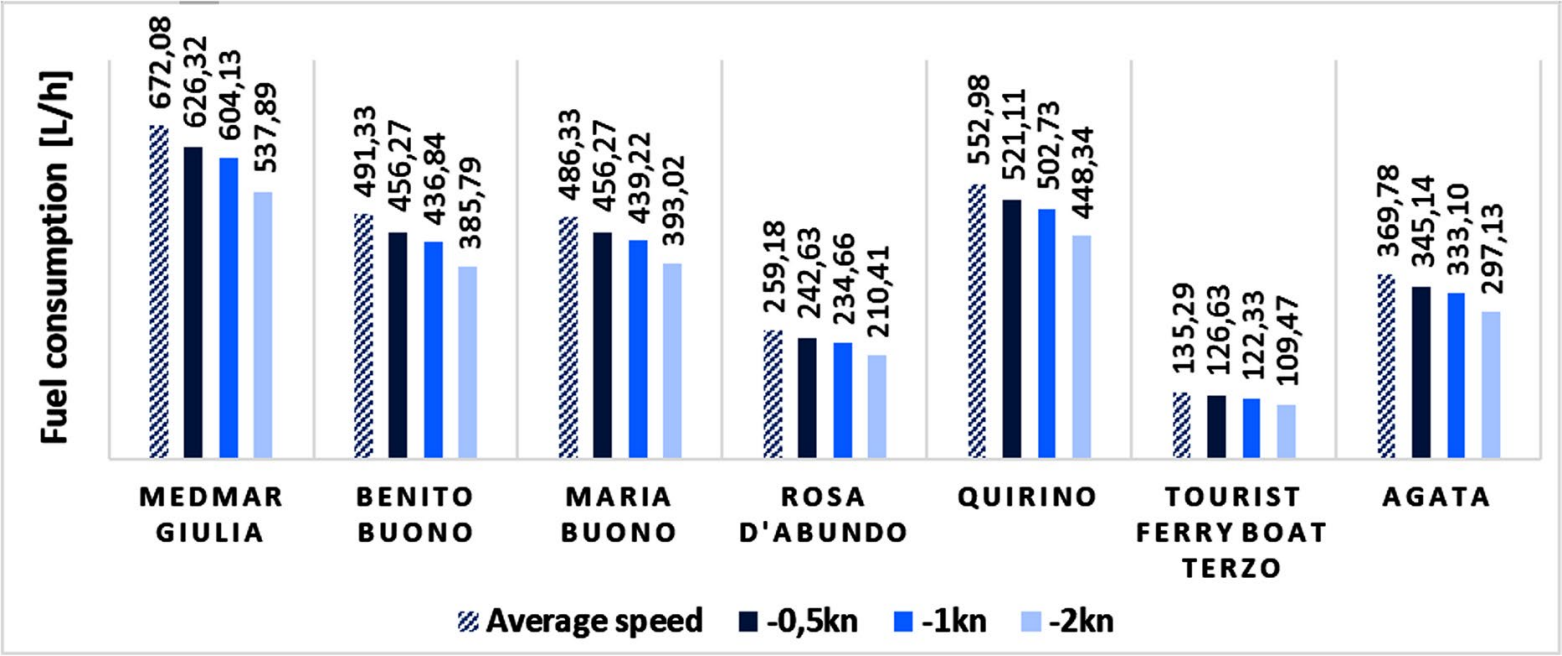
Estimated fuel consumption for each vessel at its average speed and at speeds progressively reduced by 0.5, 1 and 2 knots

Following this result, as also mentioned in the “Scenario no. 1” section, further simulations were carried out within this scenario in order to quantify the fuel savings resulting from any potential reduction in navigation speed. Figure 2 shows the variations for each vessel, starting from their average navigation speed and progressively decreasing it (−0.5 kn, −1 kn and −2 kn).

Fuel savings rates range from 6–7% for a speed decrease of 0.5 knots to 19–21% for speed decreases of 2 knots. These results are consistent with those reported in the literature for similar simulations (Corbett et al. 2009; Wiesmann 2010). Assuming as an example the ship Tourist Ferry Boat Terzo, which has an average speed of 10 kn, under the above conditions a progressive reduction in average speed of 5%, 10% and 20% was simulated for it; the corresponding decreases in fuel consumption were 6.7%, 9.6% and 19.3%. These values are coherent with what was observed in scenario 2 reported by Corbett et al. (2009), in which they simulated the dependence of CO_2_ equivalent emissions on varying speed and fuel prices, as well as on the need to satisfy the carriage of a specific amount of goods.

Predictably, since fuel consumption is very strongly correlated with emissions (*ρ* > 0.99), the observed fuel savings were proportionally reflected also in the emissions of pollutants into the atmosphere, which saw a reduction of about 6% when the average speed decreased by 0.5 knots up to 19% for decreases of 2 knots.

#### Scenarios no. 2 and no. 3

**Fig. 3:**
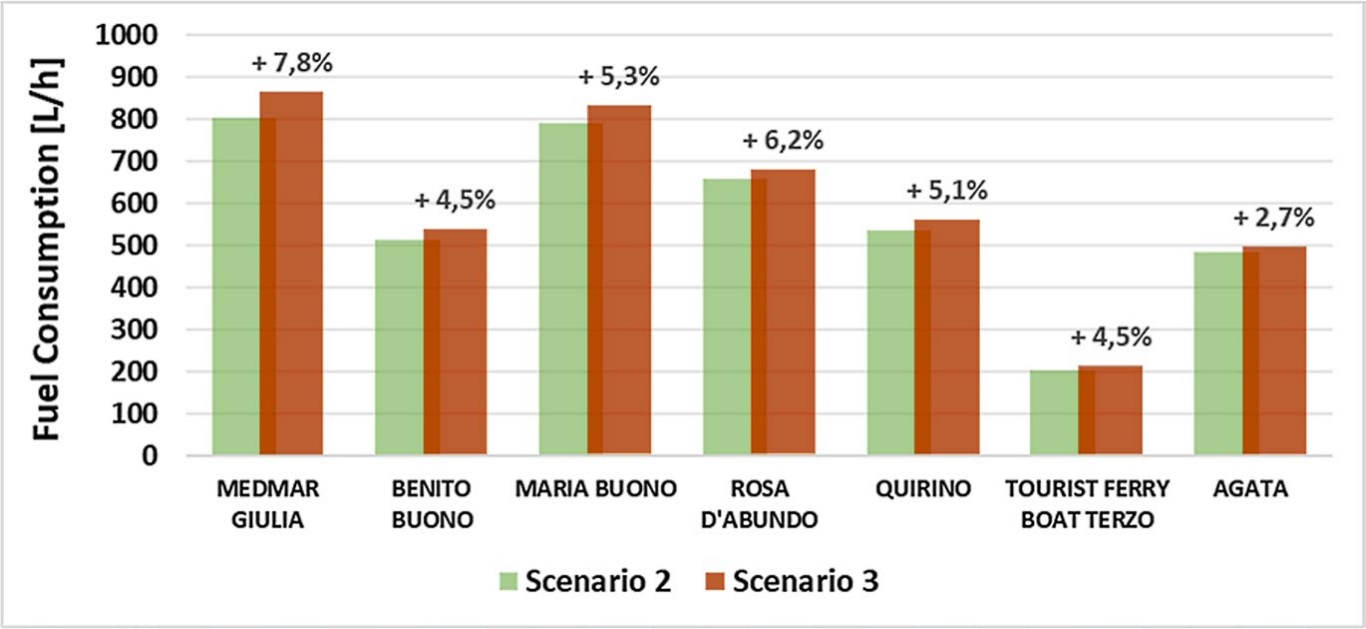
Fuel consumption simulated for each vessel within scenarios no. 2 and no. 3

Referring to the source data used in the simulations and summarised in Table 2, the first aspect that should be considered is the different percentage of cargo for each of the ships in scenario no. 2 and scenario no. 3 compared to the maximum DT shown in Table 1 for each vessel. The comparison with the maximum DT showed that, for almost all vessels, the cargo levels ranged between 50 and 60% in scenario no. 2 and 70–80% in scenario no. 3. Among the 7 ships, the vessels Giulia and Agata stand out as particular examples; specifically, the vessel Giulia goes from 70% in scenario 2 to 95% in scenario 3, while Agata goes from 46 to 54%. The reasons for this difference compared to the other vessels were their specific maximum DTs (Giulia: 711t to Agata: 1088t), and the maximum amount of cargo chosen for embarkation. Regarding the consumption, Figure 3 shows the fuel consumption of each vessel simulated with the parameters pertaining to scenarios no. 2 and no. 3.

In Fig. 3, the percentages on the bars for scenario no. 3 represent the increase in fuel consumption compared to scenario no. 2. For each of the vessels, the trend in consumption followed the increase in transported cargo, with an average increase in fuel consumption of about 5% among all vessels. But the proportionality of these increases was not linear, as other factors related to the different characteristics of individual vessels were involved, such as the power of the main engines installed, and the levels of maximum DT embarked for each vessel. An example of the dependence on this latter aspect was provided by the Giulia and Agata ships, for which the differences from the other five vessels in the fleet were already mentioned. The increase in consumption for the Giulia was higher than for the other vessels due to the fact that it was loaded (in scenario no. 3) at almost 95%, while the increase for the Agata was lower, being it loaded in scenario no. 3 at 54% of its maximum DT. When analysing pollutants emissions, their percentage increase followed that of consumptions, as a consequence of the direct dependence between the two parameters.

### Consumptions and emissions of the auxiliary services (diesel/generators)

With the information provided by the company, it was possible to calculate the effective power in kW of the DGs for each of the vessels (Table 5). The calculation was carried out for the entire fleet, by multiplying the number of DGs used by their nominal power and by the percentage of loaded cargo to which they were entitled. This calculation was made not only for each route, but also considering both phases of navigation and port docking (Table 5). Moreover, since the company provided information on the measured total consumption of DG compartments for each vessel, it was possible to calculate the consumption of the generators in litres per hour for both phases of the vessels (navigation and port docking), after which this consumption was appropriately converted into MWh with the aim of calculating the relative CO_2_ equivalent emissions (Table 5).

**Table 5:** Power actually used by DG units during navigation and port docking, with relative total consumption (navigation + port docking) expressed both in terms of fuel consumed (L/h) and associated energy (MWh). “DG consumption per route (L/h)” are real values effectively measured by MedMar technical staff.

**Name of the ship**	**DG power (kW)** port docking	**DG power (kW)** navigation	**DG consumption per route (L/h)** port docking + navigation	**DG consumption per route (MWh)** port docking + navigation
**MedMar Giulia**	196	448	288	2.862
**Benito Buono**	397	794	216	2.146
**Maria Buono**	356	712	210	2.087
**Rosa D’Abundo**	138	275	210	2.087
**Quirino**	538	806	126	1.252
**Tourist Ferry Boat Terzo**	48	96	60	0.596
**Agata**	84	168	165	1.640

Starting from the real data collected during stage (i) (see the “Overview of the methodology used for data analysis” section), the total CO_2_ equivalent emissions (t/hour) related to DG equipment were calculated, for each vessel, in the following conditions: (i) use of DG during both phases of navigation and port docking (in Table 6, “total emissions”); (ii) during the navigation phase only; (iii) during the phase of port docking only; and (iv) considering a port docking time of 40 minutes for each vessel. Table 6 shows the results obtained under the assumed conditions.

**Table 6:** DG emissions under different conditions.

**Name of the ship**	**Total emissions** **[tCO**_**2**_**/h]**	**Total emissions in navigation** **[tCO**_**2**_**/h]**	**Total emissions in port docking** **[tCO**_**2**_**/h]**	**Total emissions in port docking for 40 min** **[tCO**_**2**_**/40 min]**
**MedMar Giulia**	0.764	0.535	0.229	0.151
**Benito Buono**	0.573	0.401	0.172	0.113
**Maria Buono**	0.557	0.390	0.167	0.110
**Rosa D’Abundo**	0.557	0.390	0.167	0.110
**Quirino**	0.334	0.234	0.100	0.066
**Tourist Ferry Boat Terzo**	0.159	0.111	0.048	0.032
**Agata**	0.438	0.306	0.131	0.087

When studying the correlations between the different parameters, a moderate correlation emerged between total emissions and the number of generators (0.37 < *ρ* < 0.45) and similarly between total emissions and the real power of DG equipment on the ships (0.28 < *ρ* < 0.32). It is also worth noting that, on an hourly basis, port docking emissions account for about 30% of total emissions, while about 70% of consumption and related emissions in the DG sector occur during navigation.

### General survey on total emissions per working day and overall observations

**Fig. 4:**
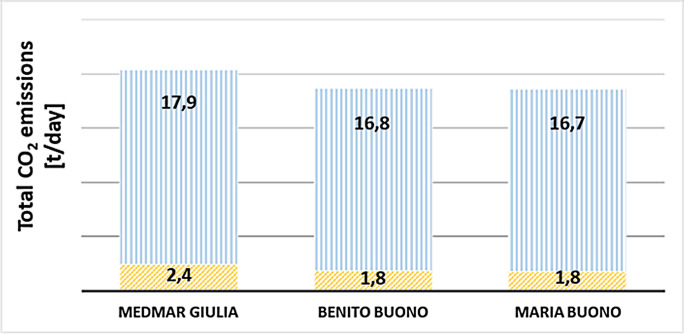
Total CO_2_ equivalent emissions over an entire working day for the three vessels: MedMar Giulia, Benito Buono and Maria Buono. Oblique bars indicate emissions during port docking phase, while solid bars indicate emissions during navigation phase

In an effort to obtain a more comprehensive description of the energetic behaviour of the vessels and to investigate in more detail the CO_2_ equivalent emissions attributable to the two different compartments (propulsion vs. auxiliary services), we simulated a real day of operation. For this calculation the following 3 vessels were considered: M. Giulia, B. Buono, M. Buono. Since MedMar vessels complete 8 navigation routes of about 60 min each in one day, and that each route involves two stops in port of about 40 min, a total of 10,56 h of “port docking” were considered for a full working day. During the stationing in port, since the vessel is in a resting condition, most of the consumption is related to the activation of auxiliary services, rather than to the propulsive system. After combining the emission shares of the propulsion and DG units, and comparing the total values obtained for the two conditions of navigation and port docking, the results obtained from this further simulation (as shown in Fig. 4) indicated that emissions during port docking were responsible for about 10–11% of the total CO_2_ equivalent emissions of a single vessel per working day. It is worth noting that this result was achieved in a uniform pattern across the fleet, despite the intrinsic differences between vessels that were highlighted in previous scenarios (note, for example, the differences observed for the MedMar Giulia vessel).

These results suggest the possibility of intervening with solutions that could mitigate pollution levels also and especially during the stationing of vessels in the port area, whose ecosystem is already severely affected (Mercogliano et al. 2016; Sciarrillo et al. 2020). Interventions which could enable the self-supply of auxiliary services (e.g. on-board batteries charged by renewable sources in the port) or efficiency improvements on engines and sailing conditions (e.g. hull cleaning and new engines) could allow to reduce a non-negligible share of the total emissions of the ship fleet, in all its operating conditions. Molland et al. (2014) identified as one of the most promising strategies for reducing the propulsive power of ships (and consequently also their consumption and emissions) and the optimisation of the hull-propeller-ruder interaction in the propulsion systems, although they emphasised that the greatest savings were still linked to the optimisation of operational strategies, i.e. not only the navigation speed investigated in the present study, but also, for example, selecting the most effective meteorological route. Xing et al. (2020), in analysing the state of the art of measures that could be adopted to reduce CO_2_ equivalent emissions from maritime transport, emphasised the implementation of environmentally friendly fuels and alternative energy sources. However, the step towards their concrete adoption is constrained by the fact that they are not universally applicable in a standardised way, because of the many differences between vessel types, their intended uses and the routes covered. For these reasons, applying diversified solutions according to shipping contexts is certainly one of the challenges that stakeholders will face in the race to reduce the environmental impacts of medium and long-distance maritime transport.

### Externalities

Externalities are positive or negative effects produced by companies through economic activity, which impact on the community or other companies as additional external revenues or costs. By applying this definition to the operational context of MedMar fleet, and following the indications given by Van Essen et al. (2019), we calculated the costs that three different types of transport (ship, car, bus) generate on the community, in order to establish comparisons. To be more representative, the following intermediate conditions were assumed: (i) non-hybrid 1800 cc petrol-powered cars with 2 passengers; (ii) bus equipped with a Euro 4 engine and 40 passengers and (iii) as a ship, Benito Buono vessel with a load of 600 passengers.

**Fig. 5:**
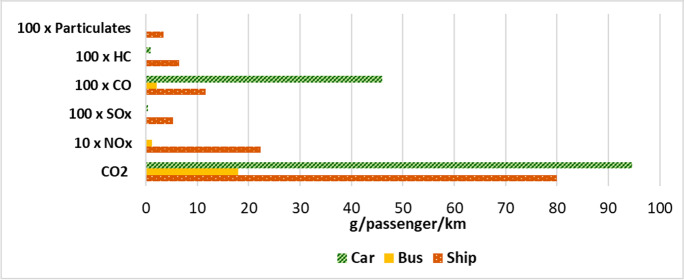
Estimated emissions for three types of transport, namely, car, bus and ship. Assumptions for the three categories are reported in the “Externalities” section.

**Fig. 6:**
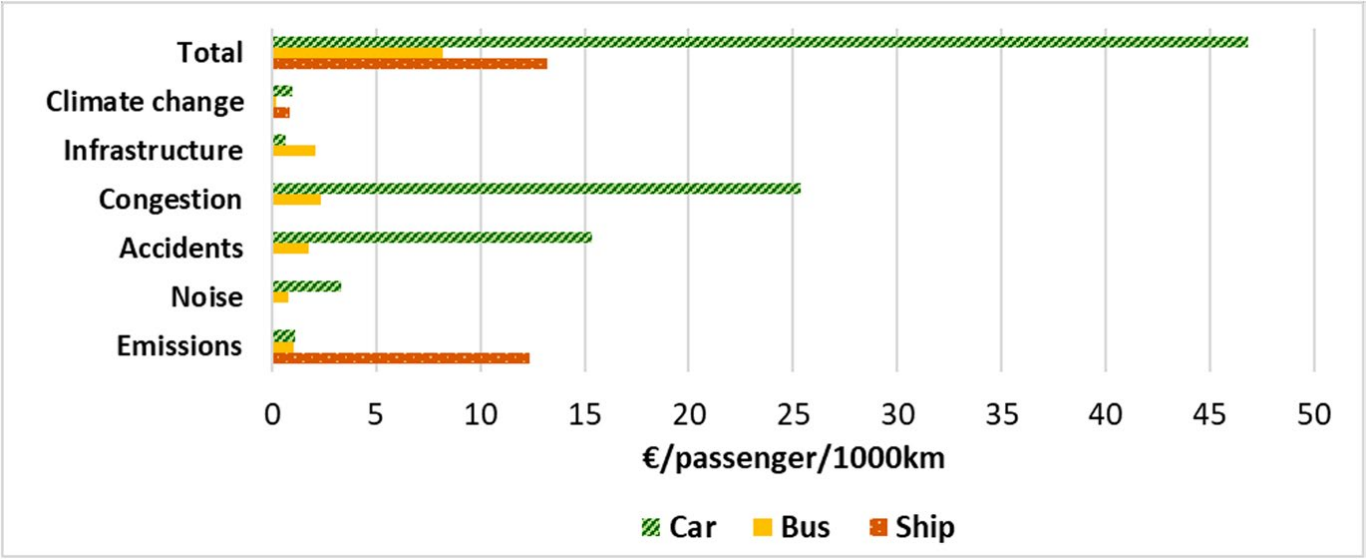
Public costs induced by three different types of transport (car, bus, ship) and for the following externalities: climate change, infrastructure, road congestion, accidents, noise and emissions. Assumptions for the three categories are reported in the “Externalities” section.

Initially, the pollutant emissions for each of the three transport systems were estimated and compared, as shown in the graph in Fig. 5. Subsequently, the externalities considered for simulation purposes were emissions, noise, accidents, congestion, infrastructure and climate change. The effects of these externalities, characterised by different impact potential, were converted into real “costs” falling on the community (Fig. 6), following the recommendations of Van Essen et al. (Van Essen et al. 2019).

Comparing the results obtained in terms of both emissions and externalities, it first emerged that cars were still the means of transport with the highest direct emission of carbon oxides (CO_2_ and CO) compared to the other two means of public transport. In addition, the impact of cars was also dominant from the point of view of externalities, especially in terms of congestion, potential accidents and noise generated (Fig. 6). As might be expected, this result is linked to the limited number of passengers that these vehicles can accommodate and, consequently, to the higher number of cars on the roads in order to meet the urban mobility demand of the population.

Nevertheless, ships are the public transport system with the greatest impact on the community in terms of emissions, especially of pollutants other than carbon oxides. As an example, NO_x_ emissions from ships in Fig. 5 (22.3 g/passenger/km) are almost 700 times higher than those from cars (0.03 g/passenger/km), while SO_x_ emissions from ships (5.2 g/passenger/km) are about 90 times higher than those from buses (0.1 g/passenger/km). In relation to that, an interesting aspect reported by Jonson et al. (Jonson et al. 2020) was that the rise in PM_2.5_ levels associated with maritime transport in the Mediterranean macro-area, which usually occurs during the summer months, was mainly related to sulphur emissions, and this phenomenon was particularly evident in those port areas subject to high maritime traffic, including the north-eastern Adriatic and the port areas of Marseille in France and Piraeus in Greece (Jonson et al., Jonson et al. 2020). However, for the present case study in general, it is difficult to establish consistent comparisons with data from the literature. In fact, most of the works dealing with emissions and consumption in the maritime sector focus on a type of ship that is different from the one addressed in this study, namely, cruise ships. Cruise ships not only differ from those of the MedMar fleet from an infrastructural point of view, but also as regards the order of magnitude of the gross tonnage and the overall carrying capacity of the vessel. In addition, both types of vessels also differ in the frequency of sailings, the distance covered and the time spent navigating and stationing in port. In relation to this aspect, in their aim to monitor air quality levels in the Naples port area, Murena et al. (2018) highlighted precisely the differences in terms of emissions and types of pollutants emitted for commercial vessels and cruise ships. Their analysis revealed that the NO_x_ and SO_x_ levels of cruise ships are over one order of magnitude more impactful than those of passenger ships; at the same time, since cruise ships visit the port area of the gulf of Naples much less frequently than passenger ships, the use of time-based normalisation techniques (e.g. considering annual averages) could mitigate the real impact that cruise ships exert in their period of stay and transit in a port area (Murena et al. 2018).

## Conclusions

This paper presented the results of a study on energy consumption and related emissions of the fleet of MedMar Navi S.p.A., a company operating in the gulf of Naples, in order to identify the critical aspects and potential opportunities for the company to further mitigate its environmental impact and achieve increasingly ambitious sustainability goals. Through appropriate simulations of scenarios, it was possible to highlight and quantify how the navigation speed and the cargo level of each vessel affect the energy performance of the fleet. These simulations were carried out by analysing first separately and then jointly the energy contributions related to the propulsion systems (engines) and to the generators supporting the auxiliary services. The main findings of such simulations can be summarised as follows:

1. Consumption and emissions of propulsion systems were strongly correlated with sailing speed, and reducing the average speed by 2 knots (about 3.7 km/h) could translate into fuel energy savings in the order of almost 20%, which is a significant amount.
2. In the scenarios where the speed of each vessel was set to its maximum value, it was possible to quantify the increase in fuel consumption related to the amount of cargo carried. In particular, when switching from an average transported load of 55% to an average transported load of 75% (with respect to the maximum cargo capacity), an average increase of about 5% was estimated for the fuel consumption of each vessel.
3. Simulating the fuel consumption due to generators for auxiliary services revealed that there were weak correlations between the total emissions of these generators and the number of such equipment in place as well as the effective power absorbed by each.
4. When combining the results from the two contributions of propulsion and auxiliary services, it emerged that generator-related consumptions contributed to about 10% of the total emissions generated during navigation phase for all vessels, despite their differences in terms of cargo capacity and navigation speed. Focusing on this aspect, a further opportunity for energy saving could be provided by the potential deployment on board of devices that allow to self-supply such generators or—more broadly—to support the energy demand of auxiliary services through renewable sources, thus further reducing future fuel demand.

We believe that the results of this study can be useful for discussion in several ways. First of all, it represents a first example in literature of a study conducted on an entire passenger-ship fleet to estimate its fuel consumption and emissions. Furthermore, the results obtained are practical and quantitative information that can be used not only by the surveyed shipping company, but also by other companies, to plan measures aimed at mitigating the energetic and environmental impact of their fleets, whether this involves reducing the speed of navigation or installing systems on board to self-supply auxiliary services from renewable sources. In addition, these results will serve as a baseline for the identification, through different innovative methodology (statistical analysis, machine learning model, etc.) of further energy saving and environmental impact reduction scenarios. Lastly, this work also addresses the concern expressed by international government authorities to provide and share data on vessel consumptions, emissions and navigation, as a necessary gap to be filled in order to work jointly towards increasingly compelling sustainability goals.

## Supplementary information

ESM 1 (DOCX 19 kb)
