# Supplementary material for: Characterizing passenger-ship emissions: towards improved sustainability for MedMar fleet (gulf of Naples)
Source: Energy Effic. 2022 Oct 18;15(8):55. doi: 10.1007/s12053-022-10064-7 (PMC9579613; doi:10.1007/s12053-022-10064-7)
Supplement: Supplementary file 1 — (DOCX 19 kb) [file 12053_2022_10064_MOESM1_ESM.docx]

# SUPPLEMENTARY DATA

Table S1 - Ship-specific technical information concerning: type of propulsion system, number of units installed and model of main engines and DG equipment

| **Name of the ship** | **Propulsion type** | **Main engines** | **DG** |
| --- | --- | --- | --- |
| **MEDMAR GIULIA** | Mechanical transmission with shaft line and no.2 4-blade controllable pitch propellers | - No.4 - DIESEL WARTSILA - 824 TS - 1030 kW at 750 RPM | - No.5 - DIESEL VOLVO PENTA - TD120 A 354 - 140 Kw at 1500 RPM |
| **BENITO BUONO** | Mechanical transmission with shaft line and no.2 3-blade controllable pitch propellers | - No.2 - DIESEL MAK - 8M452AK - 1176 kW at 375 RPM | - No.3 - DIESEL CUMMINS - VTA28G1 - 496 kW at 1500 RPM |
| **MARIA BUONO** | Mechanical transmission with shaft line and no.2 4-blade controllable pitch propellers | - No.2 - DIESEL DAHIATSU - 8DLM-32 - 2206 kW at 600 RPM | - No.2 - DIESEL YANMAR - S185L-ST - 445 kW at 900 RPM |
| **ROSA D'ABUNDO** | Mechanical transmission with shaft line and no.2 fixed-pitch propellers | - No.2 - DIESEL DEUTZ - SBA16M528 - 1706 kW at 900 RPM | - No.3 - DIESEL DEUTZ - BA6M816 - 172 kW at 1500 RPM |
| **QUIRINO** | Mechanical transmission with shaft line and no.2 4-blade controllable pitch propellers | - No.2 - DIESEL GMT - B230-12 - 1853 kW at 1100 RPM | - No.3 - DIESEL ISOTTA FRASCHINI - T6 354B230 - 336 kW at 1500 RPM |
| **TOURIST FERRY BOAT TERZO** | Mechanical transmission with shaft line and no.2 fixed-pitch propellers | - No.2 - DIESEL DEUTZ - SBA6M528 - 640 kW at 900 RPM | - No.2 - DIESEL PERKINS - 4.4TW2GM - 80 kW |
| **AGATA** | Mechanical transmission with shaft line and no.2 Voith - Schneider | - No.2 - DIESEL GMT - A2312SS - 1492 kW at 1000 RPM | - No.2 DIESEL AIFO - 821-SM - 140 kW |
